# The Impact of Egg Consumption on Gastrointestinal Health: A Systematic Literature Review and Meta-Analysis

**DOI:** 10.3390/nu17132059

**Published:** 2025-06-20

**Authors:** Nessmah Sultan, Caroline J. Tuck, Edellyne Cheng, Nicole J. Kellow, Jessica R. Biesiekierski

**Affiliations:** 1 Department of Nutrition, Dietetics & Food, Monash University, Notting Hill, VIC 3168, Australia; nessmah.sultan@monash.edu (N.S.);; 2 Department of Allied Health, Swinburne University of Technology, Hawthorn, VIC 3122, Australia; 3 Human Nutrition Group, School of Agriculture, Food and Ecosystem Science, The University of Melbourne, Parkville, VIC 3010, Australia

**Keywords:** eggs, gastrointestinal, microbiome, inflammation, TMAO

## Abstract

Objective: Eggs are a valuable source of nutrients and bioactive compounds that may influence the gastrointestinal tract by modulating the microbiome, promoting the production of gastrointestinal-related metabolites, and mediating inflammation. Limited human studies have explored the effects of whole egg intake on indices of gastrointestinal health. This systematic literature review aimed to synthesise research investigating the impact of whole egg consumption on markers of gastrointestinal health. Methods: Five databases were searched from inception until July 2024. Studies were included if they examined the link between whole egg consumption and gastrointestinal markers, including symptoms, gut microbiome composition, inflammation, colonic fermentation, and egg-derived metabolites such as trimethylamine N-oxide (TMAO) in healthy adults. Two reviewers independently conducted title and abstract and full-text screening, with conflicts resolved by a third reviewer. Similarly, two authors conducted data extraction, which was verified by a third. A risk of bias assessment was conducted using validated tools. Random effects meta-analyses were performed to summarise the effect of egg consumption on TMAO, choline, and C-reactive protein (CRP). Results: Twenty-two studies were included in a narrative synthesis and ten in the meta-analyses. Nine were randomised controlled trials (RCTs), three were non-randomised intervention trials, eight were cross-sectional, and two were prospective cohort studies. Meta-analyses indicated that egg consumption did not impact plasma TMAO *(n* = 6, *p* = 0.22) or CRP (*n* = 3, *p* = 0.45) concentrations but did increase plasma choline (*n* = 5, *p* < 0.001) in the short term (≤4 weeks). Four studies found correlations between habitual egg consumption and specific gut bacteria, although results varied as egg consumption was both positively and negatively associated with butyrate-producing genera. Conclusions: This review found conflicting results regarding egg consumption and most gastrointestinal outcomes, highlighting that future studies are needed to explore links between habitual egg intake and plasma TMAO, microbial diversity, and inflammation (PROSPERO registration: 408532).

## 1. Introduction

Eggs have remained a staple of the human diet for centuries. They are nutrient-dense, providing protein, fat, vitamins, minerals, and carotenoids [1]. Historically, the association between egg consumption and heart health has been controversial, with the high cholesterol content of the yolk thought to increase cardiovascular disease risk [2]. However, recent research has negated this claim, with several high-quality clinical trials finding that egg consumption does not increase plasma cholesterol concentrations [3,4] except in people who are hyper-responders to dietary cholesterol and may, in contrast, reduce cardiovascular disease risk through increasing plasma high-density lipoproteins [3,4,5]. Given the long-standing debate about the link between egg consumption and cardiovascular disease, research on the health effects of egg intake beyond plasma cholesterol levels remains limited. Notably, studies investigating the impact of egg consumption on gastrointestinal health are underexplored.

Eggs contain several nutrients that may have an impact on the gut, including via the microbiome, modulation of inflammation, and production of gastrointestinal-related biomarkers. Eggs are high in protein and fat, containing 6.29 g and 5 g, respectively, per medium-sized egg [6]. Studies in animal models have shown positive associations between protein and fat intake and microbial diversity [7,8,9,10,11]. However, most research on egg consumption and the gut microbiome has been limited to animal studies, with few examining this relationship in humans.

Microbial-derived fatty acids benefit the host in several ways, including by modulating inflammation and strengthening the intestinal mucosal barrier [12]. To date, no studies have specifically explored colonic protein fermentation in the context of egg consumption. However, some have explored the digestibility of egg proteins, reporting that they are highly digestible when cooked [13,14]. Egg whites contain protease inhibitors that are deactivated when heated [15], allowing for greater absorption—94% of cooked eggs compared to 65% of raw eggs [13]. Considering the potential for egg proteins to reach the large bowel, the impact of egg consumption on the production of short-chain and branched-chain fatty acids (SCFAs and BCFAs) warrants further investigation.

The high choline content of eggs is another focus area of egg-related research. Dietary choline is metabolised by the gut microbiota to produce trimethylamine (TMA), which is then absorbed in the small intestine and oxidised to trimethylamine N-oxide (TMAO) in the liver [16]. TMAO has been linked to an increased risk of chronic disease incidence and all-cause mortality [17,18]. The precise mechanisms whereby TMAO might increase the risk of chronic disease have not been fully elucidated but may involve inflammation, as plasma TMAO has been linked to increases in the inflammatory markers tumour necrosis factor (TNF-α) and interleukin-6 (IL-6) [19,20,21]. Published data on the impact of egg consumption on TMAO has been contradictory, with some studies indicating post-prandial increases in TMAO concentrations following egg ingestion, while others have shown no effect [22].

Lastly, eggs contain antimicrobials that may exhibit antibacterial, antiviral, antiparasitic, or antifungal properties upon consumption [1,23] and may reduce pro-inflammatory biomarkers [24]. The gut microbiome itself may modulate inflammation by inducing immune responses, such as T-helper cell differentiation, in the presence of pathogens [25,26,27]. Animal studies have linked gut bacteria to the release of both pro- and anti-inflammatory mediators [11,28,29]. However, the impact of eggs on gut inflammation in humans remains underexplored. While individual nutrients in eggs are known to influence various aspects of gastrointestinal health, research specifically addressing the impact of whole egg consumption on human gut health remains limited. Previous studies have often focused on animal models or isolated egg components, such as yolks or whites, rather than whole eggs. Moreover, there has been no prior comprehensive synthesis of human studies examining the relationship between habitual egg intake and gut health indicators such as the microbiome, inflammation, gastrointestinal metabolites, and colonic fermentation. This systematic review addresses this gap by collating and critically appraising available human evidence.

## 2. Methods

### 2.1. Eligibility Criteria, Databases, Search Strategy

This review was conducted according to the Preferred Reporting Items for Systematic Reviews (PRISMA) guidelines [30] and was registered with the Prospective Register of Systematic Reviews (PROSPERO) in March 2023 (408532).

The PICOS framework used to define the research question and inclusion and exclusion criteria is outlined in Table 1. Five databases (Ovid Medline, Embase, CINAHL Plus, SCOPUS, and PsychInfo) were searched from inception until April 2023. This search was repeated in July 2024 to retrieve articles published between April 2023 and July 2024. Studies were included if they examined the link between whole chicken egg consumption and gastrointestinal health in adults without chronic disease (aged > 18). Indices of gastrointestinal health were defined as any outcomes related to gastrointestinal factors, including symptoms, microbiome composition, and function (inflammation, colonic fermentation, and TMAO). Reviews and case studies were excluded. Studies were excluded if they were conducted on animals, children, or humans with chronic disease or if they involved an intervention of non-whole eggs (for example, by providing egg whites or egg yolks only without examining the impact of whole egg consumption).

A librarian was consulted to advise on the search strategy. The search terms used were as follows: (egg* adj3 ingest* or intake or eat* or consum*) AND (exp. Gastrointestinal Tract OR exp. Gastrointestinal Microbiome OR exp. Digestive System OR protein fermentation OR gastrointestinal tract OR GI tract OR gastrointestinal symptom* OR GI symptom* OR gastrointestinal sensation OR GI sensation OR gastrointestinal system OR GI system OR gastrointestinal microbi* OR GI microbi* OR gut microbi* OR motility OR emptying OR malabsorption OR inflammatory response OR cytokine concentration OR bacterial taxonomy OR bacterial abundance OR bacteria diversity OR bacterial composition). The proximity operator adj3 was used to identify terms within three words of each other (e.g., ‘egg’ within three words of ‘ingest’, ‘intake’, ‘eat’, or ‘consume’). The search was limited to studies in human participants and published in the English language. Studies were also found through hand-searching the reference lists of relevant articles. An example of the full search strategy used is presented in Supplementary Table S1.

### 2.2. Study Selection and Data Extraction

All references found during the search were uploaded to EndNote (version X9, Clarivate Analytics). Once duplicates were removed, the remaining studies were imported into Covidence for screening (Covidence Systematic Review Software, Veritas Health Innovation, Melbourne, Australia. Available at www.covidence.org (accessed on 16 March 2023)). Two independent reviewers (N.S. and J.R.B.) conducted title and abstract screening, followed by full-text screening. Conflicts that arose were resolved by discussion and a third reviewer (C.J.T.).

Data extraction was conducted after full-text screening. The data collected included author, year of publication, country of origin, study design, sample size of participants, intervention or dietary pattern assessed, outcomes examined, outcome measures, key results, and funding source. Two authors conducted the data extraction (N.S. and E.C.), which was verified by a third (J.R.B., N.J.K., and C.J.T.).

### 2.3. Risk of Bias Assessment

A risk of bias assessment was conducted using one of four tools depending on the study design, in duplicate by two authors independently (N.S. and N.J.K.). Conflicts of risk of bias classification were resolved through discussion. Randomised controlled trials (RCTs) were assessed using the Cochrane Risk-of-Bias 2 (RoB 2) tool [31]. The RoB 2 tool examines bias in five domains: method of randomisation, deviation from interventions, incomplete outcome data, outcome measurement, and selection of reported results. The risk of bias was classified as ‘low risk’, ‘some concerns’, or ‘high risk’. Non-randomised intervention trials were assessed using the Cochrane Risk Of Bias In Non-Randomized Studies - of Interventions (ROBINS-I) tool [32]. This tool identifies potential bias based on seven domains, examining confounding factors, participant selection, classification of interventions, deviation from interventions, missing data, outcome measurement, and selection of reported results. The risk of bias for each non-randomised study was classified as ‘no information’, ‘low risk’, ‘moderate risk’, ‘serious risk’, or ‘critical risk’. Prospective cohort studies were assessed using the Cochrane Risk Of Bias In Non-Randomized Studies - of Exposures (ROBINS-E) tool [33], which similarly identifies bias based on seven domains, examining confounding factors, measurement of exposure, selection of participants, post-exposure interventions, missing data, outcome measurement, and selection of supported results. Like ROBINS-I, the risk of bias for each non-randomised study was classified as ‘no information’, ‘low risk’, ‘moderate risk’, ‘serious risk’, or ‘critical risk’. Lastly, cross-sectional studies were assessed using the Joanna Briggs Institute Critical Appraisal Checklist for Cross-Sectional Studies (JBI) [34], which measures potential bias in eight domains, including were the inclusion criteria clearly defined, were the study subjects and setting described in detail, was the exposure measured in a valid and reliable way, were objective standard criteria used for measurement of the condition, were confounding factors identified, were strategies to deal with confounding factors stated, were the outcomes measured in a valid and reliable way, and was appropriate statistical analysis used. The risk of bias for each domain was classified as either ‘yes’, ‘no’, ‘unclear’, or ‘not applicable’. ‘Yes’ responses denote a lower risk of bias.

### 2.4. Data Synthesis

RCTs that examined the impact of egg consumption on gastrointestinal outcomes using similar measures (plasma TMAO, plasma choline, CRP) were subjected to random effects model meta-analyses using Review Manager (RevMan, Version 5.1. The Nordic Cochrane Centre, The Cochrane Collaboration, 2014, Copenhagen, Denmark). Using endpoint data (mean and standard deviation) from control and intervention groups, standardised mean differences (SMDs) were determined for each outcome with 95% confidence intervals (CIs). Where outcome data were not provided in the original research article, corresponding authors were contacted by email and data was requested. Where outcomes were published in graphical form, and corresponding authors failed to respond to requests, data were extracted using WebPlotDigitizer (version 4.7, https://automeris.io/WebPlotDigitizer/ (accessed on 19 September 2023)), a freely available validated web-based software program [35]. The results were combined for each outcome, and data were tested for interstudy heterogeneity using the Cochrane Q statistic and quantified by the *I*^2^ statistic with *p* < 0.05. An *I*^2^ ≥ 50% was considered substantial heterogeneity. A sensitivity analysis was conducted by omitting one study at a time to investigate the influence of a single study on each outcome estimate and heterogeneity. Non-randomised studies and RCTs without accessible, relevant outcome data were synthesised narratively.

## 3. Results

### 3.1. Study Selection

**Figure 1:**
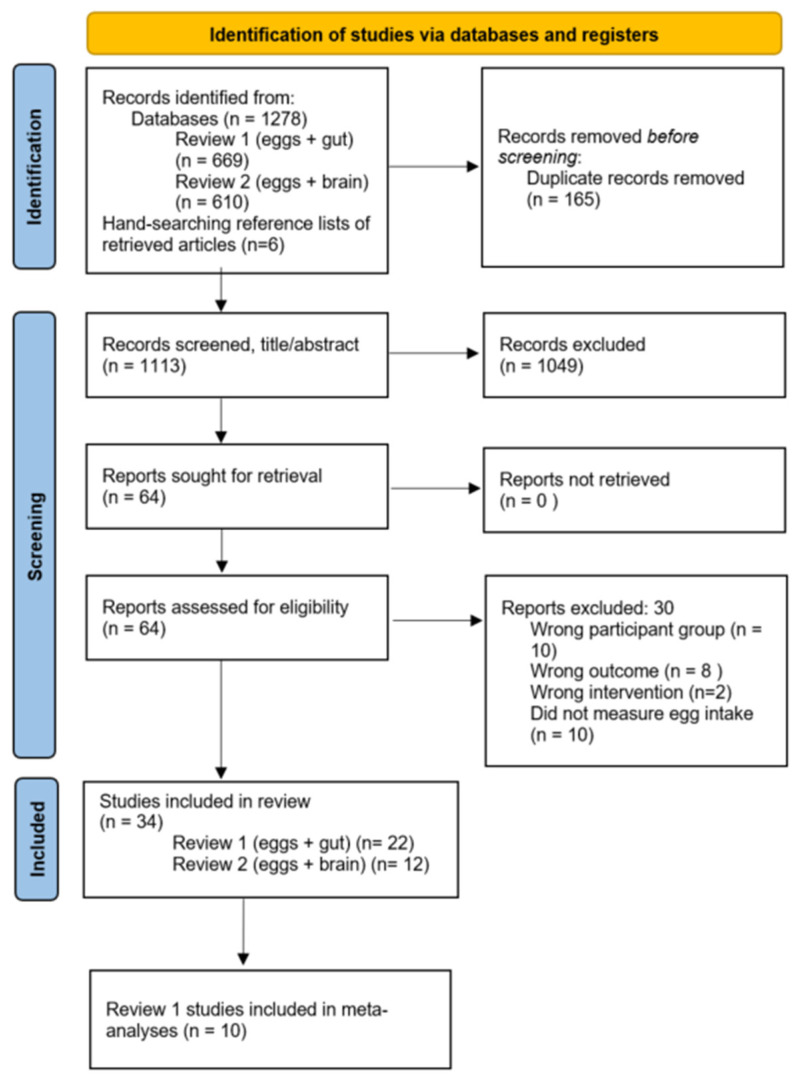
PRISMA flow diagram of the study selection. Review 1 studies included in the narrative synthesis (*n* = 22) and in the meta-analysis (*n* = 10).

This review originally explored the impact of egg consumption on both gastrointestinal health and brain function; however, based on the large number and scope of studies found, this work was subsequently split into two separate reviews. The focus of the current review, therefore, explores the association between egg consumption and gastrointestinal health only. As shown in the PRISMA diagram in Figure 1, database searching (including the original search from inception to April 2023 and the follow-up search from April 2023 to July 2024) found 1278 studies related to gastrointestinal health, while hand-searching found 6 articles. Following the removal of duplicates and studies that did not meet the inclusion criteria, 22 studies were included in the qualitative analysis and 10 in the meta-analyses.

### 3.2. Study Characteristics

The characteristics of the included studies are described in Table 2. Studies were published between 1998 and 2024 and recruited a total of 39,909 participants (participant numbers per study ranged from 6 to 32,166). Participants were aged between 18 and 85 years, and less than half (*n* = 16,011, 40%) were males. The majority were conducted in the USA (*n* = 15), followed by the UK *(n* = 2), South Korea (*n* = 1), Europe (*n* = 1), Asia (*n* = 1), Croatia (*n* = 1), Germany (*n* = 1), Poland (*n* = 1), Italy (*n* = 1), and New Zealand (*n* = 1). In terms of study design, nine RCTs were included, followed by eight cross-sectional studies, two prospective cohort trials, and three non-randomised intervention trials. For the intervention trials, the interventions ranged in dose from 2 to 4 whole eggs/day and duration from 6 h to 12 weeks. For the cross-sectional and cohort studies, the tools used to assess egg intake included 24-h food recalls, food frequency questionnaires (FFQs), and 3–7-day diet diaries. Most studies measured TMAO concentrations (*n* = 16), followed by plasma choline (*n* = 12), gut microbiome composition and diversity (*n* = 8), circulating inflammatory markers (*n* = 5), and faecal SCFAs (*n* = 1). No studies explored other relevant outcomes, such as gut symptoms.

### 3.3. Risk of Bias Across Studies

**Figure 2:**
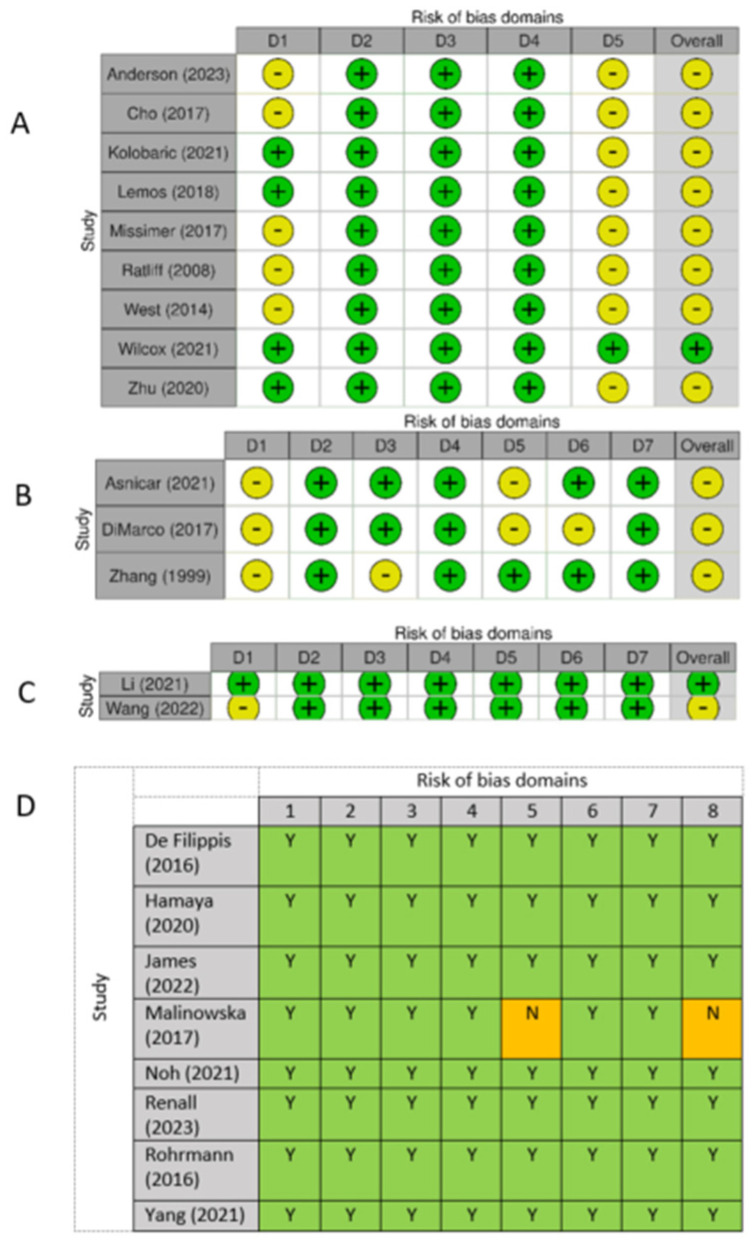
Risk of bias assessments. (**A**) RoB-2 [36,37,38,39,40,41,42,43,44]. (**B**) ROBINS-I [4,55,56]. (**C**) ROBINS-E [53,54]. (**D**) JBI [45,46,47,48,49,50,51,52]. For RoB-2: D1, Bias arising from the randomisation process; D2, Bias due to deviations from intended intervention; D3, Bias due to missing outcome data; D4, Bias in measurement of the outcome; D5, Bias in selection of reported result. For ROBINS-I: D1, Bias due to confounding; D2, Bias due to selection of participants; D3, Bias in classification of interventions; D4, Bias due to deviations from intended interventions; D5, Bias due to missing data; D6, Bias in measurement of outcomes; D7, Bias in selection of reported result. For ROBINS-E: D1, Bias due to confounding; D2, Bias arising from measurement of exposure; D3, Bias in selection of participants into the study (or into the analysis); D4, Bias due to post-exposure interventions; D5, Bias due to missing data; D6, Bias arising from measurement of the outcome; D7, Bias in selection of reported result. For JBI: 1, Were the criteria for inclusion in the sample clearly defined; 2, Were the study subjects and the setting described in detail; 3, Was the exposure measured in a valid and reliable way; 4, Were objective, standard criteria used for measurement of the condition; 5, Were confounding factors identified; 6, Were strategies to deal with confounding factors stated; 7. Were the outcomes measured in a valid and reliable way; 8, Was appropriate statistical analysis used. Green “+/Y” = low risk of bias, yellow “−/N” = moderate risk of bias. RoB 2.0, Cochrane Risk of Bias 2.0; ROBINS-I, Risk Of Bias In Non-Randomized Studies—Of Interventions; ROBINS-E, Risk Of Bias In Non-Randomized Studies—Of Exposures; JBI, Joanna Briggs Institute Critical Appraisal Checklist for Analytical Cross-Sectional Studies.

As shown in Figure 2, most studies were rated as having ‘some concerns’ or ‘moderate’ risk of bias (*n* = 13). One RCT received a ‘low’ risk of bias rating [43], as did eight observational studies [45,46,47,49,50,51,52,53]. The main sources of potential bias included failure to report the randomisation processes, failure to adjust for confounding relating to participant selection, and lack of reporting on whether outcome assessors were blinded. The funding sources of studies are described in Supplementary Table S2. Mostly, funding sources were free from bias. Seven studies were funded by the USA-based Egg Nutrition Center, which had no influence on the publication or interpretation of results.

### 3.4. Synthesis of Results

#### 3.4.1. TMAO

Sixteen studies explored the impact of egg consumption on plasma (*n* = 15) or urine (*n* = 3) TMAO [4,36,37,39,40,42,43,44,46,47,48,51,53,54,56]. The majority (*n* = 13) found no significant associations between egg consumption and blood or urinary TMAO concentrations [4,36,37,39,40,42,43,44,47,48,51,54,56]. In an RCT involving 82 healthy participants, Wilcox et al. [43] reported that both plasma and urinary TMAO significantly increased four weeks after consuming a combination of four eggs/day and a 1000 mg choline supplement, or the 1000 mg choline supplements alone, but not after consuming four eggs/day only. One cross-sectional study involving 32,166 healthy adults found that higher habitual egg intake (one egg/day) as measured by FFQs was positively associated with plasma TMAO (β = 0.081, *p* = 0.033) [52]; however, this study pooled data from 16 separate international prospective cohort studies, introducing heterogeneity to the sample. Only 14 of these 16 included studies measured dietary intake, though each used a different FFQ to assess dietary intake, and they measured TMAO concentrations through differing methods (including targeted assays or non-targeted assays). Moreover, one post-prandial study involving 40 volunteers reported significantly higher urinary TMAO six hours after consuming three whole eggs, although the plasma levels of TMAO were unchanged [37]. One prospective cohort study [53] found that more frequent habitual egg intake was linked to higher plasma TMAO according to validated FFQs (median intake of 0.43 eggs/day, *p* < 0.05) but not 7-day food records (median intake of 0.39 eggs/day). The validated FFQ constituted 152 items and asked participants how often they consumed each food in the preceding year.

**Figure 3:**
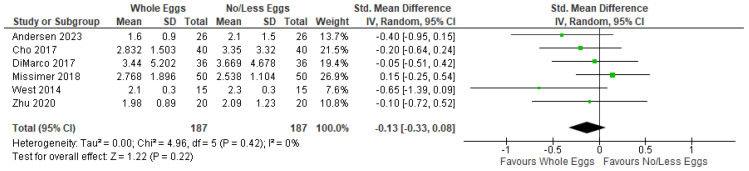
Random effects model meta-analysis for *n* = 6 studies demonstrating the relationship between whole egg intake vs. controls on plasma TMAO concentrations. Standardised Mean Difference (95% CI) shown for individual and pooled trials [4,36,37,40,42,44].

Six intervention trials that examined plasma TMAO in response to whole egg intake, compared to no/fewer eggs, were included in a random effects meta-analysis (Figure 3), which did not find significant increases in TMAO following egg consumption (SMD = −0.13; 95% CI: −0.33, 0.08; *I*^2^ = 0%; *p* = 0.22).

#### 3.4.2. Plasma Choline

Twelve articles studied plasma or serum choline in relation to egg consumption [4,36,39,40,42,43,44,48,51,54]. All the intervention trials (*n* = 7) found that an increased egg intake increased blood choline levels post-intervention [4,36,39,40,42,43,44]. Similarly, one cross-sectional study that included 122 elderly females reported an association between egg intake and circulating choline (relative difference (RD) based on high versus low egg intake = 8.2, *p* < 0.05) [48], as did a cohort study involving 3931 older adults (Spearman correlation 0.88) [54]. Conversely, a prospective cohort study by Li et al. [53] found that plasma choline was not impacted by habitual egg intake in 307 male health professionals. Likewise, a cross-sectional study of 271 German adults did not find any association between egg intake (assessed via 24-h dietary recalls) and plasma choline [51].

**Figure 4:**
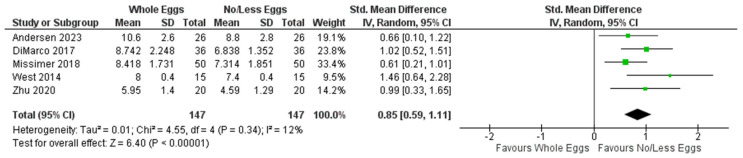
Random effects model meta-analysis for *n* = 6 studies demonstrating the relationship between whole egg intake vs. controls on plasma choline concentrations. Standardised Mean Difference (95% CI) shown for individual and pooled trials [4,36,40,42,44].

Figure 4 presents the meta-analysis findings of five intervention trials that examined plasma choline in response to whole egg intake vs. no/fewer eggs. As shown, studies collectively reported significantly higher plasma choline after consuming whole eggs compared to no/fewer eggs (SMD: 0.85; 95% CI: 0.59, 1.11; *I*^2^ = 12%; *p* < 0.00001).

#### 3.4.3. Gut Microbiome Composition and Diversity

Six studies explored links between egg consumption and microbial diversity [44,45,49,50,53,55]. Studies used either 16S rRNA (*n* = 3) or shotgun metagenomic sequencing (*n* = 3) to identify changes in gut bacterial composition. Most reported analysing both α and β diversity [49,50,55], but some reported only α diversity [44,45] or β diversity [53]. Alpha diversity was measured using the Shannon index (*n* = 3), Chao1 abundance (*n* = 1), Faith’s phylogenetic diversity (*n* = 1), Pielou’s index (*n* = 1), and observed species (*n* = 1). Beta diversity was measured using the Bray–Curtis dissimilarity (*n* = 4), Jaccard index (*n* = 1), and weighted and unweighted UniFrac distances (*n* = 1).

One RCT exploring the impact of egg consumption on gut microbiome composition and diversity in overweight postmenopausal women did not find any significant associations. This RCT was of short duration (4 weeks) and analysed the microbiome using 16S rRNA sequencing and α diversity using the Shannon index [44]. A cross-sectional study of 222 healthy adults also found no correlation between gut microbial composition or diversity and habitual egg intake, measured by a 106-item FFQ of dietary intake in the year preceding the study [49]. However, three studies [50,53,55] that explored patterns of microbiome composition and diversity in relation to long-term habitual egg intake, measured mostly via FFQs, found significant associations, although there were inconsistencies between study results (Table 3).

Three of the studies that found an association between egg intake and microbiome composition utilised shotgun metagenomic sequencing, with varying findings. Li et al. [53], in a cross-sectional study of 307 male health professionals, found that higher habitual egg intake was positively linked to *Bifidobacterium* spp. (specifically *Bifidobacterium bifidum*). Conversely, both Asnicar et al. [55] (*n* = 1098 healthy adults) and Renall et al. [50] (*n* = 286 healthy women) reported egg intake to be negatively associated with *Bifidobacterium* spp. (reduced *Bifidobacterium adolescentis* was found in both studies). The latter two studies also similarly reported a positive association between egg intake and *Eubacterium* spp. [50,55]. Regarding microbial diversity, one study reported that individuals with greater habitual egg intake displayed higher α diversity via Pielou’s index scores [50].

#### 3.4.4. Inflammatory Markers

Three RCTs (all with a moderate risk of bias) examined alterations in C-reactive protein (CRP) in response to egg consumption, with one finding that eggs significantly reduced CRP in 28 overweight men [41]. Two RCTs found CRP to be unchanged by eggs in healthy young adults [36,40].

Several RCTs examined changes in cytokines in response to consuming regular whole eggs. Most looked only at pro-inflammatory cytokines, including IL-6 (*n* = 2), TNF-α (*n* = 2), MCP-1 (*n* = 2), IL-8 (*n* = 1), TGF-β1 (*n* = 1), and IL-17A (*n* = 1). Two RCTs on healthy adults noted that eggs did not impact IL-6 [38,40]. Conversely, eggs had no impact on TNF-α concentrations in overweight men [41], but consuming two eggs per day significantly reduced TNF-α in healthy adults when compared to consuming oatmeal [40]. Neither of the RCTs that explored the impact of egg intake on MCP-1 found significant results [38,41]. Additionally, IL-8 [41], TGF-β1, and IL-17A [38] were all unchanged by egg consumption. Kolobarić et al. [38] examined the anti-inflammatory IL-10 in healthy adults but reported it was also unaffected by the consumption of three whole eggs/day for three weeks. One RCT [38] explored levels of T lymphocytes in 40 healthy young adults after three weeks of consuming three eggs/day, finding that Th17 and Tregs were significantly reduced.

**Figure 5:**
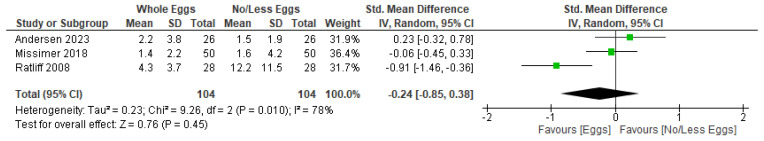
Random effects model meta-analysis for *n* = 6 studies demonstrating the relationship between whole egg intake vs. controls on plasma CRP concentrations. Standardised Mean Difference (95% CI) shown for individual and pooled trials [36,40,41].

Random effects meta-analysis results for three studies (Figure 5) were insignificant for circulating CRP concentrations following egg consumption (SMD: −0.24; 95% CI: −0.85, 0.38; *I*^2^ = 78%, *p* = 0.45). A sensitivity analysis involving the systematic removal of each study one by one revealed that data from Ratliff et al. [41] was the cause of the high heterogeneity. After removing this study from the analysis, heterogeneity was reduced to *I*^2^ = 0%, but the results of the analysis were still non-significant (*p* = 0.81).

#### 3.4.5. Faecal Short-Chain Fatty Acids

Only one included study measured the correlation between egg consumption and faecal SCFAs in a cross-sectional cohort of 153 healthy adults [45]. No significant associations were found between egg intake and SCFAs. This study utilised one 7-day weighed food record to determine egg intake and measured faecal SCFAs using three homogenised stool samples collected over consecutive weeks.

## 4. Discussion

This systematic review and meta-analysis synthesised studies that examined the effects of egg intake on markers of gastrointestinal health, including TMAO, plasma choline, gut microbial diversity, inflammatory markers, and SCFAs. Across studies, egg consumption was positively associated with plasma choline; however, the results regarding other outcomes of interest were inconclusive.

Plasma TMAO was found to be unchanged after short-term egg consumption (≤4 weeks) but was positively correlated with more frequent longer-term egg consumption (higher habitual egg intake over one year). TMAO is a prothrombotic compound that has been implicated in several chronic diseases. Egg-associated TMAO production appears to vary between individuals and is dependent on gut microbial composition (for the conversion of choline to TMA), liver FMO3 activity (which converts TMA to TMAO), and renal function (for the excretion of TMAO) [57]. Urinary TMAO was linked to a greater incidence of coronary heart disease in a study of over 100,000 people in China that controlled for diabetes, hypertension, and dyslipidaemia but not renal function [18]. Likewise, in over 5000 people, those with the highest quartile of plasma TMAO had an 86% increased risk of all-cause mortality compared to those in the lowest quartile, even when controlled for diabetes, cancer, cardiovascular disease, age, and smoking, but not when controlled for eGFR [17]. While it should be noted that fasting plasma TMAO levels are a better predictor for potential health outcomes than urinary TMAO, as TMAO is excreted renally within 6–24 h after absorption [58,59], these studies highlight a potential correlation between TMAO and incidence of disease. Therefore, the dietary prevention of increased plasma TMAO levels should be explored through further long-term cohort studies in healthy populations. The mineral present in eggs that has been linked to TMAO is choline, with RCTs finding increased plasma and urinary TMAO levels after 1000 mg choline supplementation, the equivalent of four whole eggs [43]. Moreover, the ability to convert dietary choline to TMA, the precursor to TMAO, is dependent on the presence of gut microbes capable of producing TMA [60]. Though originally studied in mice [61,62], these findings have been confirmed in human trials. A post-prandial study of 40 volunteers found an increase in plasma TMAO <4 h after consuming a combination of two hardboiled eggs and a phosphatidylcholine supplement; however, after the same participants were given antibiotics for one week to suppress gut flora, the same intervention did not impact post-prandial plasma TMAO levels [63]. Several studies have attempted to identify which gut bacteria may be linked to TMA production [37,64,65,66]; however, there is a lack of consensus across trials, requiring further study.

This systematic review could not determine with certainty specific gut bacteria associated with egg consumption. However, two studies reported similar findings regarding egg consumption and specific genera [50,55]. While Li et al. [53] found that egg intake was positively linked to *Bifidobacterium bifidum*, Asnicar et al. [55] and Renall et al. [50] found that egg intake was negatively correlated with *Bifidobacterium adolescentis. Bifidobacterium* spp. have demonstrated beneficial effects, including by producing the SCFAs acetate and butyrate [67,68]. *Bifidobacterium* spp. also converts monosodium glutamate to the neurotransmitter γ-aminobutyric acid (GABA) [69], which maintains cytoplasmic pH homeostasis to reduce neuronal excitability. RCTs have found that synbiotic supplementation, including *Bifidobacterium* spp., reduced depression, anxiety, and stress and improved sleep quality [70,71]. Further studies should explore the impact of egg consumption on levels of *Bifidobacterium* spp. and symptoms of depression, anxiety, or stress. Secondly, Asnicar et al. [55] and Renall et al. [50] found a positive correlation between egg intake and *Eubacterium* spp., which include major butyrate producers [72,73]. *Eubacterium* spp. produce butyrate from dietary carbohydrates via glycolysis [72]. Butyrate is a ligand for several G-protein coupled receptors (GPCRs), important for immune regulation, maintenance of the gut epithelial barrier, and differentiating Tregs, IL-10, and IL-18 [74,75,76]. Butyrate also inhibits the production of pro-inflammatory cytokines and is important for maintaining the health of colonocytes [77]. This is notable, as one RCT included in this review found reductions in pro-inflammatory cytokines in participants given two eggs a day for four weeks [40]. Alternatively, a cross-sectional study [45] found a non-significant negative association between egg intake and faecal butyrate levels and between egg intake and *Eubacterium* spp. Given several studies found correlations between egg consumption and butyrate-producing bacteria or reductions in inflammation, future studies should explore the impact of egg consumption on microbial diversity and abundance.

This review largely found no significant impact of eggs on inflammatory markers in healthy participants. Notably, the RCTs that explored this outcome mostly did not conduct power or sample size calculations to guide recruitment numbers [36,40,41]; therefore, it is possible that the small sample sizes were insufficient to measure the effect. A previous meta-analysis of nine RCTs conducted on healthy adults and people with type 2 diabetes also found no association between egg consumption and inflammatory markers, including CRP, TNF-α, and IL-6 [78]. On the other hand, some egg studies have found that participants who were overweight [41] or who had metabolic syndrome [79,80] experienced a reduction in inflammatory markers after egg interventions (3 eggs/day for 4–12 weeks led to reductions in TNF-α, IL-6, and CRP) [41,79,80]. This suggests that there may be a beneficial effect of eggs in people who already have heightened inflammation, although previous RCTs involving individuals with type 2 diabetes and coronary heart disease have not found significant associations between egg intake and inflammation [81,82,83,84]. As well as any possible mediating effects through influencing the gut microbial composition as described above, eggs have the potential to influence inflammation, as they contain several bioactive compounds that can be pro-inflammatory (cholesterol) or anti-inflammatory (carotenoids, immunoglobulin Y, cystatins) [1,85]. Further studies should explore the impact of egg intake on inflammatory markers by utilising longer intervention periods and analysing highly sensitive markers of inflammation to ascertain potential subtle impacts in both healthy people and people with heightened inflammation.

This review identified several unanswered gaps in the literature and implications for future studies. Firstly, this review only identified one RCT that examined the influence of egg consumption on the gut microbiome (2 eggs/day, 4 weeks) [44]. Further RCTs are required to explore the impact of eggs on the microbiome, as a larger body of research is necessary to draw conclusions. Moreover, this RCT utilised 16S rRNA bacterial sequencing and did not find significant associations between microbial diversity and egg intake. Similarly, the three observational studies that failed to associate microbiome composition or diversity with egg intake used 16S rRNA sequencing [44,45,49], while the three studies that did find associations utilised shotgun metagenomic sequencing [50,53,55]. The two most widely used culture-free techniques to identify gut microbiota include 16S rRNA sequencing and shotgun metagenomic sequencing. While more cost-intensive, shotgun sequencing is more accurate and is able to identify more taxa at a greater resolution than 16S rRNA sequencing [86]. Therefore, shotgun sequencing could be able to better identify subtle changes in the microbiome over shorter periods of time. Future RCTs should utilise this method of analysis. Secondly, this review included three studies that found associations between specific bacteria and higher habitual egg consumption. Notably, the prospective cohort studies and the non-randomised intervention trial that found these associations only reported cross-sectional data relating to egg consumption and microbial diversity [53,55]. Since these studies noted some association between egg intake and the microbiome, it would be useful for future cohort studies to assess changes in the gut microbial composition over time and associations with changes in egg consumption. Thirdly, eight intervention trials examining plasma TMAO in response to egg intake similarly did not find significant changes in the short term (<4 weeks), while two observational studies found significant links between higher habitual egg intake (0.43–1 eggs/day) and fasting plasma TMAO when controlling for eGFR [52,53]. The two observational studies that noted higher fasting plasma TMAO [52,53] classified high intake as about one egg per day, while the four studies that did not find an association either did not define ‘high’ egg consumption [47,48] or defined their highest intake as less than one egg per day [51,54]; therefore, regularly consuming one egg per day might be associated with higher fasting plasma TMAO. However, the intervention trials involved a higher dosage of 1–4 eggs per day but did not note any impact of the eggs on plasma TMAO during their short intervention periods. This suggests that long-term egg consumption may have subtle impacts on plasma TMAO sufficient to influence cardiovascular health outcomes; however, these discrepancies may also be due to participant characteristics. The participants in the intervention trials were mostly younger adults, while the subjects in the observational studies were middle-aged and older adults. The difference in age groups may suggest age-related differences in choline metabolism and TMAO production; however, this needs to be further examined in RCTs. Future prospective studies are also required to analyse associations between egg intake and changes in plasma TMAO over time. Moreover, four observational studies utilised 24-h recalls rather than FFQs, which might not be an accurate representation of a participant’s habitual egg consumption. While they can be time-consuming and may under-report intake, FFQs are more appropriate for the measurement of long-term dietary consumption and can be readministered to measure seasonal variations in intake during prospective cohort studies [87]. Future observational studies should prioritise using more comprehensive and validated FFQs to measure habitual egg intake or use a combination of FFQs and detailed 7-day food diaries. Lastly, only one study in this review explored faecal SCFAs in relation to egg intake and did not find a significant association [45]. This cross-sectional study measured egg intake using one 7-day food record and measured faecal SCFAs using three homogenised stool samples collected in consecutive weeks; therefore, it is unclear whether egg intake at the time of each stool sample collection remained consistent or varied for participants and if this had an impact on findings. Considering that undigested egg proteins have the potential to reach the large bowel, future research should explore the effect of cooked and raw egg intake on microbial metabolites such as SCFAs and BCFAs.

This was the first systematic review and meta-analysis to explore the effect of egg consumption on gastrointestinal health. A limitation of this review was that most studies reported gastrointestinal outcomes (microbiome, inflammation, plasma TMAO) as secondary outcomes, and several of the cross-sectional studies explored the association between general dietary intake and the outcomes of interest rather than focusing specifically on egg intake. Therefore, numerical values of blood biomarker levels in relation to egg intake were mostly not reported. Additionally, the heterogeneity of dietary intake measures used across studies introduced a limitation in the ability to compare findings. Studies relying on 3–7-day food diaries or 24-h recalls alone were unable to report long-term habitual egg intake. In contrast, studies measuring longer-term intake used various FFQs, both validated and non-validated, ranging from 99 to 220 food items and differing in comprehensiveness. Furthermore, most were rated as having a moderate risk of bias due to methodological limitations such as unclear randomisation procedures, lack of blinding, and small sample sizes without power calculations. Additionally, several observational studies did not adequately adjust for key confounders such as overall dietary patterns or baseline health status. These limitations may reduce confidence in the observed associations and highlight the need for more rigorously designed future studies.

This review consistently found a positive association between egg consumption and plasma choline across studies. Most studies reported no significant effects of egg intake on plasma TMAO, microbiome diversity, inflammation, or faecal SCFAs, suggesting that regular egg consumption does not adversely affect gastrointestinal health in healthy adults. However, a small number of studies reported associations between habitual egg intake, plasma TMAO levels, and butyrate-producing bacterial genera. These findings point to potential biological effects that merit further investigation. Future research should prioritise well-powered, long-term trials using sensitive microbiome analysis techniques to clarify these relationships. Such evidence will be crucial to informing dietary guidelines and optimising recommendations for egg consumption in the context of gut health.

## Figures and Tables

**Table 1:** PICOS criteria of the included studies.

	Inclusion Criteria	Exclusion Criteria
Population	Adults without chronic disease aged 18 years and over	Animals; adults who were breastfeeding or pregnant for the duration of the study; humans aged < 18 years; adults diagnosed with any chronic disease
Intervention	Whole chicken egg consumption as a dietary intervention or measured via the collection of prospective or retrospective diet intake data using food frequency questionnaires, food records, or dietary recall	Dietary interventions that did not include whole chicken eggs, including trials that included the provision of part of an egg, such as the egg whites or egg yolks only; observational studies that did not specifically measure egg intake
Comparison	A control group that was provided with an alternative intervention that did not include whole chicken eggs or that was not provided with an intervention; in the case of observational studies, the comparison group would include participants who reported a low egg intake	A control group that did not report levels of egg consumption or a control group that consumed a similar volume of whole eggs as the intervention group
Outcome	Any outcomes related to gastrointestinal factors, including symptoms, microbiome composition, inflammation, colonic fermentation, and gastrointestinal metabolites (TMAO)	Outcomes that are unrelated to gastrointestinal factors; outcomes related to appetite or satiety
Study Type	Randomised controlled trials; Non-randomised intervention trials; observational studies (cross-sectional, prospective, and retrospective cohort studies included)	Reviews; case studies

**Table 2:** Characteristics of the included studies.

First Author (Year of Publication)	Participants (Country, No. of Participants, Mean Age (Years), Mean BMI (kg/m^2^), Gender)	Study Design			Outcomes	Sample Collection Methods	Analysis Methods	Summary of Findings
		Randomised controlled trials						
		Design	Intervention	Duration				
Andersen (2023) [36]	USA, *n* = 26, healthy young adults, 21.7 ± 4.7 years, 22.5 ± 2.8 kg/m^2^, 19% males	Crossover trial. Lead-in: 4 weeks egg-free diet Washout: 4 weeks egg-free diet Blinding: not described	Group 1: 3 whole eggs/day Group 2: 3 egg whites/day	4 weeks	a. Plasma TMAO b. Plasma choline c. High-sensitivity C-reactive protein (hsCRP)	a, b, & c. Overnight fasted blood samples pre- and post-intervention	a & b. Measured by a commercial lab (LabCorp) c. Automated multiplex assay analyser	a. ∅ b. ↑ significantly higher in 3 whole-egg group compared to egg-free washout c. ∅
Cho (2017) [37]	USA, *n* = 40, healthy males, 27.8 ± 1.0 years, 24.2 ± 0.4 kg/m^2^	Crossover trial. Lead-in: n/a Washout: 1 week Blinding: not described	Group 1: 3 whole eggs Group 2: 170 g beef steak Group 3: 170 g fish fillet Group 4: control phase, 2 packages of applesauce 180 g	6 h (post-prandial)	a. Plasma and urine TMAO b. Gut microbiome composition c. α diversity—Faith, Chao1, observed species d. β diversity—unweighted UniFrac	a. Blood and urine samples at baseline (fasting), 15 min, 30 min, 1 h, 2 h, 4 h, and 6 h after eating b, c, & d. Stool samples at baseline	a. LCMS b, c, & d. 16S rRNA gene amplicon sequencing	a. ∅ in plasma. ↑ significantly in urine at 6 h compared to baseline b, c & d. did not assess correlation with egg consumption
Kolobarić (2021) [38]	Croatia, *n* = 40, healthy young adults, 23.8 ± 2.8 years, 24.2 ± 3.0 kg/m^2^, 50% males	Parallel arm, 2 groups Blinding: double blinding	Group 1 (intervention, *n* = 19): 3 hard-boiled *n*-3 PUFA-enriched eggs/day, providing 1053 mg of *n*-3 PUFA Group 2 (control, *n* = 21): 3 hard-boiled regular eggs/day, providing 249 mg of *n*-3 PUFA	3 weeks	a. T lymphocytes (Th17, Tregs) b. Pro-inflammatory cytokines (IL-17A, IL-23, IL-6, MCP-1, TGF-β1) c. Anti-inflammatory cytokines (IL-10)	a, b, & c. Overnight fasted blood plasma in EDTA tubes pre- and post-intervention	a. Isolated from peripheral blood mononuclear cells by flow cytometry. Cell staining with mouse anti-human antibodies b & c. Multiplex assay kits	a. nTreg oocytescytes and Th17cells ↓ significantly after regular eggs and *n*-3 PUFA-enriched eggs b. TGF-β1 ↑ significantly after *n*-3 PUFA-enriched eggs but not after regular eggs ∅ in IL-6 ∅ in IL-17A ∅ in MCP-1. Results not reported for IL-23 c. ∅
Lemos (2018) [39]	USA, *n* = 29, healthy young adults, 25.6 ± 2.3 years, 24.3 ± 2.9 kg/m^2^, 47% males	Crossover trial. Lead-in: 2 weeks Washout: 3 weeks Blinding: not described	Group 1 (intervention): 3 whole eggs/day providing 400 mg choline Group 2 (control): 1.5tablets of choline bitartrate/day, providing 400 mg of choline	4 weeks	a. Plasma TMAO b. Plasma choline	a & b. Overnight fasted blood plasma in EDTA tubes pre- and post-intervention	a & b. LCMS	a. ∅ b. ↑ significantly after whole eggs intervention but not after choline supplementation
Missimer (2018) [40]	USA, *n* = 50, healthy young adults, 23.3 ± 3.1 years, 23.2 ± 2.1 kg/m^2^, 50% males	Crossover trial. Lead-in: not described Washout: 3 weeks Blinding: not described	Group 1: 2 whole eggs/day Group 2: 1 packet of oatmeal/day	4 weeks	a. Plasma TMAO b. Plasma choline c. CRP d. Cytokines (IL-6, TNF-α)	a, b, c, & d. Fasting blood samples pre- and post-intervention	a & b. LCMS c. Automated multiplex assay analyser d. Commercially available assay kits	a. ∅ b. ↑ after eggs compared to oatmeal c. ∅ d. IL6 ∅. TNF-α ↓ significantly after eggs compared to oatmeal
Ratliff (2008) [41]	USA, *n* = 28, overweight males (BMI 26–37 kg/m^2^), aged 40–70, mean age and mean BMI not reported	Parallel arm, 2 groups Blinding: single blinding (participants)	All participants placed on the same carbohydrate-restricted diet (% energy from CHO:fat:protein = 17:57:26) Group 1 (intervention, n = 15): liquid whole eggs, 640 mg cholesterol per day, equivalent to 3–4 eggs/day Group 2 (placebo control, n = 13): liquid fat-free eggs	12 weeks	a. CRP b. Cytokines (TNF-α, IL-8, MCP-1)	a & b. Overnight fasted blood plasma in EDTA tubes, pre- and post-intervention	a & b. Multiplex assay kits	a. ↓ significantly in whole-egg group. Non-significant ↑ in fat-free egg group b. ∅ in TNF-α ∅ IL-8 MCP-1 significantly ↓ in fat-free egg group only, ∅ in whole-egg group
West (2014) [42]	USA, *n* = 15, lacto-ovo vegetarian females of reproductive age, 35.7 ± 12.9 years, 23.7 ± 4.7 kg/m^2^	Crossover trial. Lead-in: 2 weeks Washout: 4 weeks Blinding: single blinding (participants)	Group 1: 6 *n*-3 fatty acids-enriched eggs/week Group 2: 6 non-enriched eggs/week Group 3: egg-free control phase, walnuts consumed in place of eggs	4 weeks	a. Plasma TMAO b. Plasma choline	a & b. Fasted blood samples pre- and post-intervention	a & b. LCMS	a. ∅ b. ↑ significantly in groups 1 and 2 compared to control (group 3). Non-significant between groups 1 and 2.
Wilcox (2021) [43]	USA, *n* = 82, healthy adults, 28 (24.0–38.8) years, 36.9 (22.8–31.9) kg/m^2^, 42% males	Parallel arm, 5 groups Blinding: single blinding (researcher)	Group 1 (n = 18): 4 whole hard-boiled eggs Group 2 (n = 20): 2 500 mg choline bitartrate tablets Group 3 (n = 16): 4 whole hard-boiled eggs and 2 500 mg choline bitartrate tablets Group 4 (n = 18): 4 hard-boiled egg whites and 2 500 mg choline bitartrate tablets Group 5 (n = 10): 6 420 mg phosphatidylcholine tablets	4 weeks	a. Plasma TMAO b. Plasma choline c. 24-h urinary TMAO and spot urine TMAO	a & b. 8 h fasted bloods taken weekly, results reported pre- and post-intervention c. Three 24-h urine samples collected. Spot urine collected weekly, results reported pre- and post-intervention	a & b. LCMS	a. ↑ in groups 2, 3, 4 ∅ in groups 1, 5 b. ↑ in all groups c. 24 h urine ↑ in groups 2, 3, 4 ∅ in groups 1, 5 Spot urine ↑ in group 2, ∅ in groups 1, 3, 4, 5
Zhu (2020) [44]	USA, *n* = 20, overweight postmenopausal females with hypercholesterolaemia, 57.7 ± 5.6 years, 28.3 ± 3.0 kg/m^2^	Crossover trial. Lead-in: 2 weeks Washout: 4 weeks Blinding: not described	Group 1 (intervention): 100 g liquid whole egg Group 2 (control): 100 g liquid egg whites	4 weeks	a. Plasma TMAO b. Plasma choline c. Gut microbiome composition d. α diversity—Shannon	a & b. Overnight fasted blood plasma in EDTA tubes pre- and post-intervention c & d. Stool samples collected with uBiome kits pre- and post-intervention	a & b. LCMS c & d. 16S rRNA gene amplicon sequencing	a. ∅ b. ↑ after whole-egg intervention c & d. ∅
		Cross-sectional trials						
		Participant data sources/recruitment	Measurement of egg intake					
De Filippis (2016) [45]	Italy, *n* = 153, healthy adults (*n* = 51 vegetarians, *n* = 51 vegans, *n* = 51 omnivores), 39 ± 9 years, 21.9 ± 2.5 kg/m^2^, 42% males	Participants recruited from 4 geographically distanced cities in Italy (Bari, Bologna, Parma, Torino). Vegan and vegetarians recruited with collation of the Italian Society of Vegetarian Nutrition.	7-day weighted food diaries Egg intake categorised as: High Mediterranean diet adherence: 0 g eggs/day Low Mediterranean diet adherence: 9.7 g eggs/day		a. Gut microbiome composition b. α diversity—weighted and unweighted UniFrac distance c. Faecal short-chain fatty acids (SCFAs)	a, b, & c. Three stool samples collected on the same day of 3 consecutive weeks and were homogenised	a & b. 16S rRNA gene amplicon sequencing c. GC-MS/SPME	a. Non-significant but stronger positive association between eggs and Adlercreutzia and Coriobacteriaceae and negative association with Eubacterium and Lachnospiraceae c. Non-significant but stronger negative association between eggs and butanoate and propyl acetate
Hamaya (2020) [46]	USA, *n* = 620, healthy middle-aged adult males, 67.7 ± 7.7 years, 25.5 (23.6–28.0) kg/m^2^	2011–2012 Men’s Lifestyle Validation Study	1. Two 152-item FFQs completed at baseline and 6 months. Median (IQR) of egg intake according to FFQs: 0.43 (0.1–0.9) eggs/day 2. Two 7-day food diaries, completed at baseline and 6 months. Median (IQR) of egg intake according to 7-day food diaries: 0.39 (0.17–0.68) eggs/day		a. Plasma TMAO	a. Two fasted blood plasma samples 6 months apart	a. LCMS	a. ↑ significant positive association with egg intake when using FFQ, but not when using 7-day food diary
James (2022) [47]	USA, *n* = 361, healthy adults, 39.9 (13.3) years, 27.0 (4.7) kg/m^2^, 48% males (age and BMI reported for n = 120 participants of TMAO tertile 2 as average age and BMI of total cohort not reported)	The Nutritional Phenotyping Study 2015–2019	Three 24-h dietary recalls. Two on weekdays, one on a weekend. Categories of egg intake not defined. The evening prior to the study visit, they consumed a standardised meal, providing 280.7 mg of choline (included 80 g of eggs).		a. Plasma TMAO b. Plasma choline c. CRP d. Cytokines (TNF-α, IL-6) e. Gut microbiome composition f. α diversity—Shannon, Pielou’s evenness, observed species	a, b, c, & d. fasted blood plasma samples e & f. stool samples collected using sanitary collection supplies	a & b. LCMS c & d. Multiplex assay kits e & f. 16S rRNA gene amplicon sequencing	a. ∅ based on egg consumption b, c, d, e, & f. did not assess correlation with egg consumption
Malinowska (2017) [48]	Poland, *n* = 122, healthy elderly females, 68.5 ± 7.4 years, 26.7 ± 4.1 kg/m^2^	Participants recruited from the University of the Third Age and a public nursing home	90 item-FFQ Categories of egg intake not defined.		a. Plasma TMA and TMAO b. Plasma-free choline	a & b. fasted blood plasma samples	a & b. LCMS	a. ∅ based on egg consumption b. ↑ significant positive association with egg intake
Noh (2021) [49]	South Korea, *n*= 222, healthy adults, 29.6 (20–51) years, 22.9 (19.1–28.5) kg/m^2^, 49% males	National Institute of Agricultural Sciences of Korea and the International Agency for Research on Cancer (NAS-IARC) study, 2018	106-item semi-quantitative FFQ Categories of egg intake not defined.		a. Gut microbiome composition b. α diversity—Chao1, Shannon, Faith c. β diversity—Bray–Curtis, weighted and unweighted UniFrac	a, b, & c. stool samples collected on-site during the study visit. Stored in nucleic acid collection tubes.	a, b, & c. 16S rRNA gene amplicon sequencing.	a, b, & c. ∅ significant patterns noted based on egg consumption
Renall (2023) [50]	New Zealand, *n* = 286, females (*n* = 125 Pacific Islander origin, *n* = 161 European origin), 28 (22, 35) years, 28.1 (23.0, 33.4) kg/m^2^	PRedictors linking Obesity and gut MIcrobiomE (PROMISE) 2016–2017	1.5-day non-consecutive estimated food record (5DFR) 2. 220-item semi-quantitative FFQ (NZWFFQ) Egg intake of 60 g/day considered a higher intake		a. Gut microbiome composition b. α diversity—Pielou’s c. β diversity—Bray–Curtis, Jaccard	a & b. stool samples stored in participant home freezers for 11–14 days prior to storage at −80 °C	a, b, & c. Shotgun metagenomic sequencing	a, b, & c. ↑ habitual egg intake linked to microbiota profiles, including butyrate-producing species ↓ habitual egg intake linked to microbiota profile with more lactic acid producing species α diversity (Pielou’s) ↑ in individuals with higher habitual egg intake Associations between microbial composition/diversity and habitual egg intake displayed in Table 3
Rohrmann (2016) [51]	Germany, *n* = 271, healthy adults, 50 (37, 63) years, 26.1 (24.0–29.4) kg/m^2^ for males and 44 (36, 59) years, 25.2 (22.4, 28.9) kg/m^2^ for females, 38% males	2002–2003 Bavarian Food Consumption Survey	Three 24-h dietary recalls Egg intake categorised as: 0 g/day 0.1< 17 g/day ≥17 g/day		a. Plasma TMAO b. Plasma choline c. CRP d. Cytokines (TNF-α, IL-6)	a & b. non-fasted blood plasma samples	a & b. LCMS	a & b. ∅ based on egg consumption c & d. did not assess correlation with egg consumption
Yang (2021) [52]	USA, Europe, Asia, *n* = 32,166 healthy adults aged 19–84 (average age of total cohort not reported), BMI not reported, 39% males	TMAO Pooling Project. Pooled data from 16 international studies.	FFQs used in 14 studies (2 studies excluded from diet analysis) Average intake of eggs across studies: 0.2–0.8 eggs/day		a. Plasma TMAO	a. blood samples	a. Targeted and untargeted assays used across studies	a. ↑ significant positive association with egg intake (associated with 1 serving eggs/day)
		Prospective cohort trials						
		Participant data sources/recruitment	Measurement of egg intake	Duration (sample collection timepoints)				
Li (2022) [53]	USA, *n* = 307, healthy adult males (all health professionals), 71.9 years, 26.3 kg/m^2^ (only mean age and BMI reported for participants of TMAO quartile 3 as average age and BMI of total cohort not reported)	2011–2012 Men’s Lifestyle Validation Study (MLVS)	1. Two 152-item FFQs. First FFQ completed at baseline and second FFQ completed 12–13 months later. Median (IQR) of egg intake according to FFQs: 0.43 (0.14–0.93) eggs/day 2. Two 7-day food diaries. First conducted at baseline. Second at 6 months. Median (IQR) of egg intake according to 7-day food diaries: 0.4 (0.2–0.7) eggs/day	13 months (baseline, 6 months)	a. Plasma TMAO b. Plasma choline c. Gut microbiome composition d. β-diversity—Bray-Curtis	a & b. two fasted blood plasma samples taken at each timepoint, 24–72 h apart (four in total) c & d. two stool samples taken at each timepoint, 24–72 h apart (four in total)	a & b. LCMS c & d. Shotgun metagenomic sequencing	a. ↑ significant positive association with egg intake when using FFQ, but not when using 7-day food diary b. ∅ c. Associations between microbial composition and habitual egg intake displayed in Table 3 d. did not assess correlation with egg consumption
Wang (2022) [54]	USA, *n* = 3931, older adults, 72.9 ± 5.6 years, BMI not reported, 37% males	1989–2015 Cardiovascular Health Study (CHS). Participants recruited between 1989 and 1993. Followed up every 6 months until 2015.	1. Baseline 99-item picture-sort FFQ 2. Semiquantitative FFQ at follow up in 1995–1996 Average intake of eggs at baseline: 0.2 ± 0.3 eggs/day	Median study period of 12.5 years (baseline, follow up in 1996–1997)	a. Plasma TMAO b. Plasma choline	a & b. fasted blood samples taken at baseline (1989–1993) and at follow up (1996–1997)	a & b. LCMS	a. ∅ at baseline b. ↑significant positive association at baseline Follow-up data used in mediation analyses for atherosclerotic cardiovascular disease risk
		Non-randomised intervention trials						
		Participant data sources/recruitment	Intervention	Duration				
Asnicar (2021) [55]	UK and USA, *n* = 1098, healthy adults, 45.6 ± 11.9 years, 25.6 ± 5.0 kg/m^2^, 38% males in UK cohort and 41.3 years, BMI not reported, 32% males in USA cohort	Personalised Responses to Dietary Composition Trial (PREDICT) 2018–2019	Baseline study visit (day 1): participants given ‘metabolic challenge meal’ (890 kcal, 86 g CHO, 53 g fat, 16 g protein, 2 g fibre) and ‘medium fat and carb lunch meal’ (500 kcal, 71 g CHO, 22 g fat, 10 g protein, 2 g fibre) Home-phase (days 2–14): participants given standardised test meals to consume for breakfast on all days and lunch on days 2 and 3, differing in macronutrient distribution Dietary intake during trial measured via mobile phone app. Habitual egg intake measured via FFQs (different tools used in UK vs. US).	14 days	a. Gut microbiome composition b. α diversity—Shannon, observed species c. β diversity—Bray–Curtis	a. Stool samples collected at day 0 and day 14 using EasySampler stool collection kit in UK or FECOTAINER stool collection kit in USA	a. Shotgun metagenomic sequencing	a. associations between microbial composition and habitual egg intake displayed in Table 3 b & c. did not assess correlation with egg consumption
Di Marco (2017) [4]	USA, *n* = 36, healthy young adults, 24.1 ± 2.2 years, 24.3 ± 2.5 kg/m^2^, 50% males	n/a	Lead-in: 2 weeks Washout: 2 weeks Blinding: not described Group 1: 1 whole egg/day Group 2: 2 whole eggs/day Group 3: 3 whole eggs/day	4 weeks	a. Plasma TMAO b. Plasma choline	a & b. blood plasma samples, unclear if fasting samples	a & b. LCMS	a. ∅ b. ↑ significantly with increasing egg intake in a dose-dependent manner
Zhang (1998) [56]	UK, *n* = 6, healthy males, 32 ± 5 years, BMI not reported	n/a	Participants fed a specific breakfast plus 227 g of each ‘test’ food group. Forty-six foods were tested. Each test day separated by 1 week washout period. Control phase: the specific breakfast without the addition of the ‘test’ food.	8 h (post-prandial)	a. Urine TMAO	a. Urine samples collected over 0–8 h. Unclear how many urine samples were collected.	a. LCMS	a. ∅

Continuous data is presented as mean ± SD where parametric and median (IQR) where non-parametric. Only gastrointestinal-related outcomes were reported across studies. BMI, body mass index; ∅, no change; ↑, increased; ↓, reduced; TMAO, trimethylamine N-oxide; CRP, C-reactive protein; LCMS, liquid chromatography mass spectrometry; 16S rRNA, 16S ribosomal ribonucleic acid; PUFA, polyunsaturated fatty acids; IL, interleukin; TNF, tumour necrosis factor; MCP, monocyte chemoattractant protein; TGF, transforming growth factor; GC-MS/SPME, gas chromatography mass spectrometry/solid-phase microextraction; FFQ, food frequency questionnaire.

**Table 3:** Studies reporting significant correlations between microbial composition and/or diversity and egg consumption (3 out of 6 studies found no significant differences).

First Author (Year of Publication)	Study Design	Measurement of Egg Intake	Microbiota Quantification Technique	Diversity-Related Outcome (Diversity Metric)	Positively-Associated Bacteria (Genus/Species)	Negatively-Associated Bacteria (Genus/Species)
Asnicar (2021) [55]	Non-randomised intervention trial	FFQ	Shotgun metagenomic sequencing	α diversity (Shannon, observed species)—did not assess correlation with egg consumption β diversity (Bray–Curtis)—did not assess correlation with egg consumption	*Eubacterium eligens* *Firmicutes bacterium CAG:95* *Firmicutes bacterium CAG:170*	*Bifidobacterium adolescentis* *Bifidobacterium catenulatum* *Bifidobacterium longum* *Cenarchaeum symbiosum* *Clostridium bolteae CAG:59*
Li (2022) [53]	Prospective cohort trial	FFQ 7-day food diary	Shotgun metagenomic sequencing	β diversity (Bray–Curtis)—did not assess correlation with egg consumption	*Alistipes indistinctus* *Bacteroides intestinalis* *Bifidobacterium bifidum* *Streptococcus vestibularis*	*Alistipes putredinis* *Clostridium bolteae*
Renall (2023) [50]	Cross-sectional	5-day non-consecutive estimated food record 220-item semi-quantitative FFQ (NZWFFQ)	Shotgun metagenomic sequencing	α diversity (Pielou’s)—↑ in individuals with higher habitual egg intake β diversity (Bray–Curtis, Jaccard)—did not assess correlation with egg consumption	*Akkermansia muciniphila* *Alistipes putredinis* *Collinsella aerofaciens* *Coprococcus* sp. *ART55 1* *Eubacterium rectale* *Faecalibacterium prausnitzii* *Lactobacillus ruminis* *Ruminococcus bromii* *Subdoligranulum unclassified*	*Bifidobacterium adolescentis* *Bifidobacterium bifidum*

FFQ, food frequency questionnaire; ↑, increased.

## Data Availability

Data used in this review is available from the corresponding authors upon reasonable request.

## Supplementary Materials

The following supporting information can be downloaded at: https://www.mdpi.com/article/10.3390/nu17132059/s1, Supplementary Table S1: Search Strategy (MEDLINE), Supplementary Table S2: Funding Sources of Included Studies.
