# Supplementary material for: The Impact of Egg Consumption on Gastrointestinal Health: A Systematic Literature Review and Meta-Analysis
Source: Nutrients. 2025 Jun 20;17(13):2059. doi: 10.3390/nu17132059 (PMC12250686; doi:10.3390/nu17132059)
Supplement: Supplementary file 1 [file nutrients-17-02059-s001.zip › nutrients-3694426-supplementary.pdf]

Supplementary Materials

Supplemental Table S1. Search strategy (MEDLINE)

#	Query	Results from 2 Mar 2023
1	(egg* adj3 (ingest* or intake or eat* or consum*)).ti,ab,kf.	2,734
2	exp Gastrointestinal Tract/ or exp Gastrointestinal Microbiome/	715,308
3	exp Digestive System/	1,294,186
4	protein fermentation.mp. [mp=title, book title, abstract, original title, name of substance word, subject heading word, floating sub-heading word, keyword heading word, organism supplementary concept word, protocol supplementary concept word, rare disease supplementary concept word, unique identifier, synonyms, population supplementary concept word, anatomy supplementary concept word]	153
5	(gastrointestinal tract or GI tract or gastrointestinal symptom* or GI symptom* or gastrointestinal sensation or GI sensation or gastrointestinal system or GI system or gastrointestinal microbi* or gut microbi*).mp.	146,975
6	(motility or emptying or malabsorption or inflammatory response or cytokine concentration).mp. [mp=title, book title, abstract, original title, name of substance word, subject heading word, floating sub-heading word, keyword heading word, organism supplementary concept word, protocol supplementary concept word, rare disease supplementary concept word, unique identifier, synonyms, population supplementary concept word, anatomy supplementary concept word]	244,178
7	(bacterial taxonomy or bacterial abundance or bacterial diversity or bacterial composition).mp. [mp=title, book title, abstract, original title, name of substance word, subject heading word, floating sub-heading word, keyword heading word, organism supplementary concept word, protocol supplementary concept word, rare disease supplementary concept word,	8,448

	unique identifier, synonyms, population supplementary concept word, anatomy supplementary concept word]	
8	2 or 3 or 4 or 5 or 6 or 7	1,580,460
9	1 and 8	206
10	limit 9 to humans	106
11	limit 10 to animals	50
12	10 not 11	56
13	limit 12 to "review articles"	2
14	12 not 13	54
15	limit 14 to English language	48

Supplemental Table S2. Funding sources of included studies

First author (year of publication)	Funding source
Asnicar (2021)	Funded by grants from the Wellcome Trust, Medical Research Council/British Heart Foundation Ancestry, Biological Informative Markers for Stratification of Hypertension, the European Research Council, National Cancer Institute of the National Institutes of Health, the Premio Internazionale Lombardia e Ricerca 2019, Biotechnology and Biological Sciences Research Council, the European Research Council, Swedish Research Council, Novo Nordisk Foundation, Medical Research Council, European Union, Chronic Disease Research Foundation, Zoe Global and the National Institute for Health Research-funded BioResource, Clinical Research Facility and Biomedical Research Centre based at Guy's and St Thomas' NHS Foundation Trust in partnership with King's College London.
Anderson (2023)	Funded by the Agriculture and Food Research Initiative Grant from USDA
Cho (2017)	Funded by the Egg Nutrition Center
De Filippis (2016)	Funded by a grant from the Italian Ministry of University and Research
DiMarco (2017)	Funded by the Egg Nutrition Center
Hamaya (2020)	Funded by a grant from National Institute for Health Research
James (2022)	Funded in part by the Beef Checkoff, the USDA, Agricultural Research Service and the National Institute of Health
Kolobarić (2021)	Funded by European Structural and Investment Funds to Science Centre of Excellence for Personalised Health Care, the Josip Juraj Strossmayer University of Osijek, Scientific Unit for Research, Production and Medical Testing of Functional Food
Lemos (2018)	Funded by the Egg Nutrition Center and the Brazilian National Council for Scientific and Technological Development
Li (2022)	Funded by the Egg Nutrition Center
Malinowska (2017)	Funded by the National Science Centre in Poland
Missimer (2018)	Funded by the Esperance Family Foundation and the Egg Nutrition Center
Noh (2021)	Funded by a research program for Agricultural Science & Technology Development from Rural Development Administration
Ratliff (2008)	Funded by the Egg Nutrition Center

Renall (2023)	Funded by the Health Research Council of New Zealand
Rohrmann (2016)	Funded by the Bavarian Ministry of Environment, Health and Consumer Protection and the Kurt-Eberhard-Bode-Stiftung
Wang (2022)	Funded by the National Heart, Lung, and Blood Institute
West (2014)	Funded by the American Egg Board and the Agriculture Research Institute at California State Polytechnic University, Pomona
Wilcox (2021)	Funded by a grant from National Institute for Health Research and the Office of Dietary Supplements
Yang (2021)	Studies in this international pooled analysis were funded by:<br>- The National Heart, Lung, and Blood Institute<br>- The National Center for Advancing Translational Sciences<br>- The National Cancer Institute<br>- American Diabetes Association<br>- National Institutes of Health<br>- National Institute for Health Research<br>- Medical Research Council/ British Heart Foundation Ancestry<br>- Biological Informative Markers for Stratification of Hypertension<br>- Chronic Disease Research Foundation<br>- National Natural Science Foundation of China<br>- Yamagata Prefectural Government<br>- Japan Society for the Promotion of Science
Zhang (1998)	Funded by The Leverhulme Trust
Zhu (2020)	Funded by the Egg Nutrition Council and the West Coast Metabolomics Center pilot grant, and the USDA National Institute of Food and Agriculture
